# COVID-19 Cases from the First Local Outbreak of the SARS-CoV-2 B.1.1.7 Variant in China May Present More Serious Clinical Features: A Prospective, Comparative Cohort Study

**DOI:** 10.1128/spectrum.00273-21

**Published:** 2021-08-04

**Authors:** Yang Song, Ziruo Ge, Shuping Cui, Di Tian, Gang Wan, Shuangli Zhu, Xianbo Wang, Yu Wang, Xiang Zhao, Pan Xiang, Yanli Xu, Tingyu Zhang, Long Liu, Gang Liu, Yanhai Wang, Jianbo Tan, Wei Zhang, Wenbo Xu, Zhihai Chen

**Affiliations:** a Emergency Department of COVID-19, Beijing Ditan Hospital, Capital Medical University, Beijing, China; b National Institute for Viral Disease Control and Prevention, Chinese Center for Disease Control and Prevention, Beijing, China; c Peking University, Ditan Teaching Hospital, Beijing, China; Keck School of Medicine of the University of Southern California

**Keywords:** whole-genome analysis, B.1.1.7 variant, COVID-19, SARS-CoV-2, clinical features

## Abstract

The SARS-CoV-2 B.1.1.7 variant has increased sharply in numbers worldwide and is reported to be more contagious than the nonvariant. Little is known regarding the detailed clinical features of B.1.1.7 variant infection. Data on 74 COVID-19 cases from two outbreaks in two districts of Beijing, China were extracted from a cloud database, including 41 cases from Shunyi District (Shunyi B.1.470 group) and 33 from Daxing (Daxing B.1.1.7 group) from December 25, 2020 to January 17, 2021. We conducted a comparison of the clinical characteristics. Seven clinical indicators of the Daxing B.1.1.7 group were significantly higher than those of the Shunyi group, including the proportion with fever over 38°C, the levels of C-reactive protein (CRP), serum amyloid A (SAA), creatine kinase (CK), d-dimer (DD), and CD4^+^ T lymphocytes (CD4^+^ T), and the proportion with ground-glass opacity (GGO) in the lung (*P* values of ≤0.05). After adjusting for age, B.1.1.7 variant infection was a risk factor for elevated CRP (*P* = 0·045), SAA (*P* = 0·011), CK (*P* = 0·034), and CD4^+^ T (*P* = 0.029) and for the presence of GGO (*P* = 0.005). The median threshold cycle (*C_T_*) value of reverse transcriptase quantitative PCR (RT-qPCR) tests of the N gene target in the Daxing B.1.1.7 group was significantly lower (*P* = 0.036) than that in the Shunyi B.1.470 group. Clinical features, including a more serious inflammatory response, pneumonia, and a possibly higher viral load, were detected in the cases infected with B.1.1.7 SARS-CoV-2. The B.1.1.7 variant may have increased pathogenicity.

**IMPORTANCE** The SARS-CoV-2 B.1.1.7 variant, which was first identified in the United Kingdom, has increased sharply in numbers worldwide and was reported to be more contagious than the nonvariant. To our knowledge, no studies investigating the detailed clinical features of COVID-19 cases infected with the B.1.1.7 variant have been published. Local epidemics have rarely occurred in China, but occasionally, a small clustered outbreak triggered by an imported SARS-CoV-2 strain with only one chain of transmission could happen. From late 2020 to early 2021, two clustered COVID-19 outbreaks occurred in Beijing, one of which was caused by the B.1.1.7 variant. The COVID-19 patients from the two outbreaks received similar clinical tests, diagnoses, and treatments. We found that the B.1.1.7 variant infection could lead to a more serious inflammatory response, acute response process, more severe pneumonia, and probably higher viral loads. This therefore implies that the B.1.1.7 variant may have increased pathogenicity.

## INTRODUCTION

Since December 2019, the coronavirus disease-19 (COVID-19) pandemic caused by the highly infectious virus severe acute respiratory syndrome coronavirus-2 (SARS-CoV-2) has been rapidly spreading, which has posed a great threat to global public health (1, 2). With the continuous transmission and mutation of SARS-CoV-2, some viral variants of concern (VOCs) have been reported in recent months (3–8). On December 14, 2020, the United Kingdom reported a SARS-CoV-2 VOC that belonged to the PANGO lineage B.1.1.7 (9, 10), referred to as SARS-CoV-2 VOC-202012/01, B.1.1.7, 501Y.V1, or 20I/501Y.V1 (B.1.1.7 hereafter). The B.1.1.7 variant was estimated to have emerged in late September 2020 and has increased sharply to become the predominant SARS-CoV-2 strain in England, and it quickly became a global concern (7, 11, 12).

With the strict prevention and control policies implemented in China, local epidemics have rarely occurred in Beijing. Occasionally, a small clustered outbreak triggered by an imported SARS-CoV-2 strain with only one chain of transmission could happen. On Jan 17, 2021, a clustered COVID-19 outbreak in the community took place in Daxing District, Beijing. Confirmed by whole-genome sequencing and lineage typing results (9, 10), this outbreak was caused by SARS-CoV-2 B.1.1.7. This was the first local transmission of B.1.1.7 in China, which constituted a new challenge to the prevention and control of COVID-19 in China. Three weeks prior to the Daxing outbreak, another local COVID-19 outbreak occurred in Shunyi District, Beijing, caused by the lineage B.1.470 (9, 10), which was detected mostly in Asian countries. Both outbreaks were well controlled within a month.

Since the early transmission of COVID-19 in January 2020, a cohort of COVID-19 cases has been established in Beijing Ditan Hospital; since June 2020, the COVID-19 cases in Beijing have been admitted only to Ditan Hospital, after which a cloud database was established and maintained by Ditan Hospital in cooperation with a Beijing technology company (Beijing Zechuang Tiancheng Technology Development Co., Ltd.). All relevant data for COVID-19 cases are constantly being entered into the cloud database to prepare for future prospective studies. So far, the data of approximately 697 cases have been input completely. In this study, groups of two recent independent clustered outbreaks caused by distinct lineage strains and occurring in different districts in Beijing (Daxing B.1.1.7 group and Shunyi B.1.470 group) were selected as the study subjects from the cloud database. The COVID-19 cases from the two groups received similar clinical tests and treatments, and each case was observed for at least 28 days.

This study aimed to compare the clinical presentations, reverse transcriptase quantitative PCR (RT-qPCR) results, and whole-genomic features of cases from the Daxing B.1.1.7 group and Shunyi B.1.470 group to evaluate the COVID-19 severity of cases infected by the B.1.1.7 variant.

## RESULTS

### Whole-genome sequencing and analysis of SARS-CoV-2 samples in the two outbreaks.

**FIG 1:**
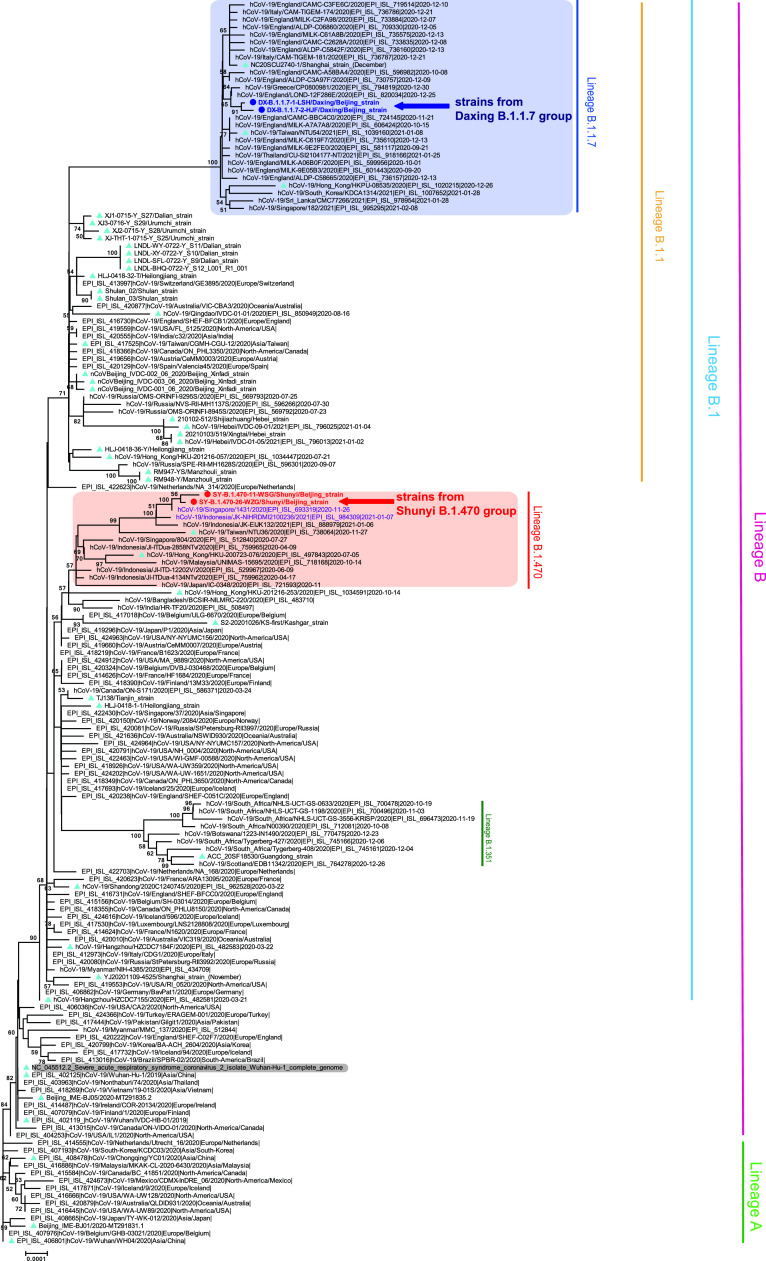
Neighbor-joining phylogenetic tree based on the whole-genome sequences of the SARS-CoV-2 representative strains. The two representative strains from the Daxing B.1.1.7 group are indicated by deep blue dots, deep blue font, and a blue left arrow, while the strains from the Shunyi B.1.470 group are indicated by red dots, red font, and a red left arrow. Strains associated with other previous outbreaks in China are indicated with ice-blue triangles. The B.1.1.7 and B.1.470 lineages are highlighted with blue and light red backgrounds, respectively, and the two strains that shared high homology with the strains of the Shunyi B.1.470 group are colored purple. The Wuhan reference strain is shaded in gray. The PANGO lineages are marked and colored on the right. The tree was rooted using strain WH04 (EPI_ISL_406801) in accordance with the root of the Pangolin tree.

Clinical samples from 8 patients of the Daxing outbreak and 13 patients of the Shunyi Outbreak were sent to the Chinese CDC for further sequencing. Some samples may be degraded due to transportation or storage. Finally, a total of 9 whole-genome sequences with a genome coverage over 98% were obtained, including 7 from the Daxing outbreak and 2 from the Shunyi outbreak. Compared with the Wuhan reference sequence (EPI_ISL_402119), seven Daxing strains shared 31 nucleotide substitutions, six strains were identical, and one strain had an additional substitution (32 substitutions) (Table 1). These seven strains shared all 28 nucleotide mutations that were first detected in the B.1.1.7 variant from the United Kingdom (Fig. 1).

**TABLE 1:** Comparison of the whole-genome features between the two groups

Feature	Data for Daxing B.1.1.7 group	Data for Shunyi B.1.470 group
No. of sequences	7	2
PANGO lineage	B.1.1.7	B.1.470
VOC or not	VOC-202012/01	Non-VOC
No. of shared nucleotide substitutions	31	24
Shared nucleotide substitution sites (reference strain Wuhan-Hu-1, EPI_ISL_402119)	C241T, C913T, C3037T, C3267T, C5388A, C5986T, T6954C, C12036G, C12747T, C12970T, C14408T, C14676T, C15279T, T16176C, A23063T, C23271A, A23403G, C23604A, C23709T, T24506G, G24914C, C27972T, G28048T, A28111G, G28280C, A28281T, T28282A, G28881A, G28882A, G28883C, C28977T	C241T, C1454G, C3037T, T6778C, C9679T, G11291A, T11296G, C14120T, C14408T, C17421T, G18315A, C18687T, C18877T, C21597T, A23403G, G25563T, G25855C, G26051T, C26681T, C26735T, G27870T, C27903T, C28887T, C29311T
Additional substitution site	T21991G	T20246G
Amino acid mutations on Spike protein	H69-V70del, Y144del, N501Y, A570D, D614G, P681H, T716I, S982A, D1118H	S12F, D614G

The two strains of the Shunyi outbreak shared 24 nucleotide substitutions, and one of them had an additional substitution (Table 1), which contained the single-nucleotide polymorphisms (SNPs) defining the PANGO lineage B.1.470 confirmed by the Pangolin COVID-19 Lineage Assigner web application (9, 10) (https://pangolin.cog-uk.io/). This lineage is composed of only one hundred more sequences in the GISAID database, most of which (77.0%) were from Indonesia. Strains from the GISAID database (13) that had high homology with these 2 strains were retrieved, including a Singaporean and an Indonesian strain (Fig. 1).

### Comparison of the general information and basic clinical manifestations in the two groups.

Of the 33 cases from the Daxing B.1.1.7 group and the 41 cases from the Shunyi B.1.470 group (Table 1), male cases accounted for 45.50% and 61.00%, respectively. The median age of the two groups (39 years [interquartile range {IQR} 30.50 to 62.50] versus 31 years [IQR 27.50 to 41.00]) showed a statistically significant difference (*P* = 0.014). Moderate cases accounted for the highest percentage of both groups (66.67% versus 48.78%). The most common symptom in both groups was fever, followed by dry cough and dry throat/pharyngeal discomfort. Of patients infected with the B.1.1.7 variant, 14 (46.43%) had fever over 38°C, a significantly higher proportion than the 9 (21.95%) patients observed infected with the B.1.470 nonvariant (*P* = 0.015).

### Comparison of laboratory tests, imageological diagnoses, and treatment measures.

There were no significant differences in the levels of white blood cells, neutrophils, lymphocytes, platelets, alanine aminotransferase, or aspartate aminotransferase between the two groups (Table 2). However, the levels of C-reactive protein (CRP), serum amyloid A (SAA), creatine kinase (CK), and d-dimer (DD) in the Daxing B.1.1.7 group were significantly higher than those in the Shunyi B.1.470 group (*P* = 0.005, 0.003, 0.040, and 0.038, respectively). When we excluded the 1 patient in the Daxing B.1.1.7 group and the 2 patients in the Shunyi B.1.470 group who were not tested for T lymphocytes due to their young age, the abnormal proportion of CD4^+^ T lymphocytes (CD4^+^ T) in the Daxing B.1.1.7 group was significantly higher than that in the Shunyi B.1.470 group (*P* = 0.003). For the imaging diagnosis, there were no significant differences in the incidence of pneumonia, but ground-glass opacity (GGO) was observed significantly more often in the B.1.1.7 group (*P* = 0.001). The antibody detection results and clinical treatments were not significantly different between the two groups.

**TABLE 2:** Comparison of the general situation and clinical manifestations

Variable	Value(s) for Daxing B.1.1.7 group (*n* = 33)	Value(s) for Shunyi B.1.470 group (*n* = 41)	*P* value
Male (*n* [%])	15 (45.50)	25 (61.00)	0.183
Average age (range)	39.00 (30.50, 62.50)	31.00 (27.50, 41.00)	0.014
Average days from onset to admission (range)	1.00 (1.00, 2.00)	1.00 (1.00, 3.00)	0.133
Average body mass index (range)	23.11 (20.84, 25.08)	22.48 (20.45, 25.57)	0.898
Clinical severity level (*n* [%])			
Asymptomatic	4 (12.12)	8 (19.51)	0.391
Mild	7 (21.21)	13 (31.70)	0.312
Moderate	22 (66.67)	20 (48.78)	0.123
Severe	0 (0.00)	0 (0.00)	
Critical	0 (0.00)	0 (0.00)	
Underlying disease (*n* [%])	13 (39.39)	14 (34.15)	0.641
Hypertension	3 (9.09)	6 (14.63)	0.723
Diabetes	2 (6.06)	3 (7.32)	>0.999
Clinical manifestations (*n* [%])			
Fever	19 (57.58)	19 (46.34)	0.337
Temp ≥ 38°C	14 (46.43)	9 (21.95)	0.015
Intolerance of cold	2 (6.06)	0 (0.00)	0.195
Dry cough	14 (42.42)	17 (41.46)	0.934
Expectoration	5 (15.15)	4 (9.76)	0.361
Nasal congestion	4 (12.12)	2 (4.88)	0.397
Runny nose	1 (3.03)	2 (4.88)	>0.999
Hyposmia	1 (3.03)	1 (2.44)	>0.999
Hypogeusia	0 (0.00)	1 (2.44)	>0.999
Dry throat/pharyngeal discomfort	6 (18.18)	16 (39.02)	0.051
Sore throat	4 (12.12)	3 (7.32)	0.693
Headache	1 (3.03)	3 (7.32)	0.624
Asthenia	5 (15.15)	1 (2.44)	0.083
Dizziness	2 (6.06)	0 (0.00)	0.195
Muscle soreness	3 (9.09)	3 (7.32)	>0.999
Joint pain	1 (3.03)	0 (0.00)	0.446
Shortness of breath	0 (0.00)	0 (0.00)	>0.999
Dyspnea	0 (0.00)	0 (0.00)	>0.999
Chest tightness	2 (6.06)	2 (4.88)	>0.999
Chest pain	0 (0.00)	0 (0.00)	>0.999
Conjunctival hyperemia	0 (0.00)	0 (0.00)	>0.999
Nausea	0 (0.00)	2 (4.88)	0.499
Vomiting	0 (0.00)	0 (0.00)	>0.999
Diarrhea	1 (3.03)	2 (4.88)	>0.999
Abdominal pain	0 (0.00)	0 (0.00)	>0.999

### B.1.1.7 variant infection is the main risk factor for more serious COVID-19 clinical features.

In order to avoid the influence of older age in the Daxing B.1.1.7 group, binary logistic regression analysis was further performed to investigate the levels of CRP (<7 mg/liter or ≥7 mg/liter), SAA (<10 mg/liter or ≥10 mg/liter), CK (<150 U/liter or ≥150 U/liter), DD (<0.5 mg/liter or ≥0.5 mg/liter), CD4^+^ T (<700 mg/liter or ≥706 mg/liter), and GGO in the lung (Table 3). After adjusting for age, we found that the group factor (B.1.1.7 variant infection or nonvariant infection) was the main risk factor for higher CRP (odds ratio [OR] = 2.79, *P* = 0.045), higher SAA (OR = 5.03, *P* = 0.011), higher CK (OR = 0.22, *P* = 0.034), GGO (OR = 5.42, *P* = 0.005), and higher CD4^+^ T (OR = 3.31, *P* = 0.029).

**TABLE 3:** Comparison of laboratory tests, *C_t_* results, and treatment measures

Variable	Values for Daxing B.1.1.7 group (*n* = 33)	Values for Shunyi B.1.470 group (*n* = 41)	*P* value
Laboratory tests			
White blood cell count (×10^9^/liter)	5.16 (4.32, 7.15)	5.69 (4.49, 7.02)	0.628
Neutrophil count (×10^9^/liter)	3.23 (2.60, 4.55)	3.45 (2.44, 4.64)	0.948
Neutrophil percentage (%)	65.30 (54.42, 71.25)	62.30 (56.90, 72.96)	0.786
Lymphocyte count (×10^9^/liter)	1.42 (0.93, 1.91)	1.39 (0.91, 1.82)	0.996
Lymphocyte percentage (%)	23.44 (19.25, 34.25)	27.40 (18.02, 35.05)	0.732
Platelet count (×10^9^/liter)	223.00 (185.00, 262.50)	217.40 (181.50, 269.00)	0.716
C-reactive protein (mg/liter)	4.30 (2.45, 12.15)	1.80, (0.85, 4.95)	0.005
Serum amyloid A (mg/liter)	21.50 (12.50, 50.70)	12.00 (5.20, 26.95)	0.003
Alanine aminotransferase (U/liter)	16.60 (11.15, 28.50)	21.00 (12.55, 37.80)	0.370
Aspartate aminotransferase (U/liter)	26.30 (18.90, 34.15)	23.20 (17.85, 29.45)	0.236
Lactic dehydrogenase (U/liter)	187.50 (170.05, 228.15)	194.90 (174.25, 224.80)	0.708
Creatine kinase (U/liter)	110.50 (53.15, 152.40)	70.40 (54.35, 103.05)	0.040
Prothrombin time (s)	12.20 (11.60, 12.70)	12.20 (11.45, 12.80)	0.947
Activated partial thromboplastin time (s)	33.50 (30.80, 35.10)	31.75 (29.50, 34.35)	0.257
d-dimer (mg/liter)	0.31 (0.20, 0.48)	0.24 (0.17, 0.31)	0.038
T lymphocyte (cells/μl)			
<1027	14.00 (43.75)	21.00 (53.85)	0.397
≥1027	18.00 (56.25)	18.00 (46.15)	
CD4^+^ T lymphocyte (cells/μl)			0.003
<706	21.00 (65.63)	12.00 (30.77)	
≥706	11.00 (34.38)	27.00 (69.23)	
CD8^+^ T lymphocyte (cells/μl)			
<320	15.00 (46.88)	17.00 (43.60)	0.782
≥320	17.00 (53.13)	22.00 (56.41)	
Imaging examination (*n*, [%])			
Pneumonia unilateral	10.00 (30.30)	15.00 (36.59)	0.570
Pneumonia	15.00 (45.45)	11.00 (26.83)	0.095
Ground-glass opacity	15.00 (45.45)	5.00 (12.20)	0.001
Antibody detection (*n*, [%])			
IgM (+)	1.00 (3.03)	2.00 (4.88)	>0.999
IgG (+)	1.00 (3.03)	3.00 (7.32)	>0.999
Treatments (*n*, [%])			
Favilavir	7.00 (21.21)	4.00 (9.76)	0.295
Favilavir + Arbidol	1.00 (3.03)	4.00 (9.76)	0.373
Interferon	1.00 (3.03)	1.00 (2.44)	>0.999
Traditional Chinese medicine	23.00 (69.70)	27.00 (65.85)	0.726
Oxygen inhalation	5.00 (15.15)	8.00 (19.51)	0.624

### Comparison of RT-qPCR threshold-crossing values between the two groups.

**FIG 2:**
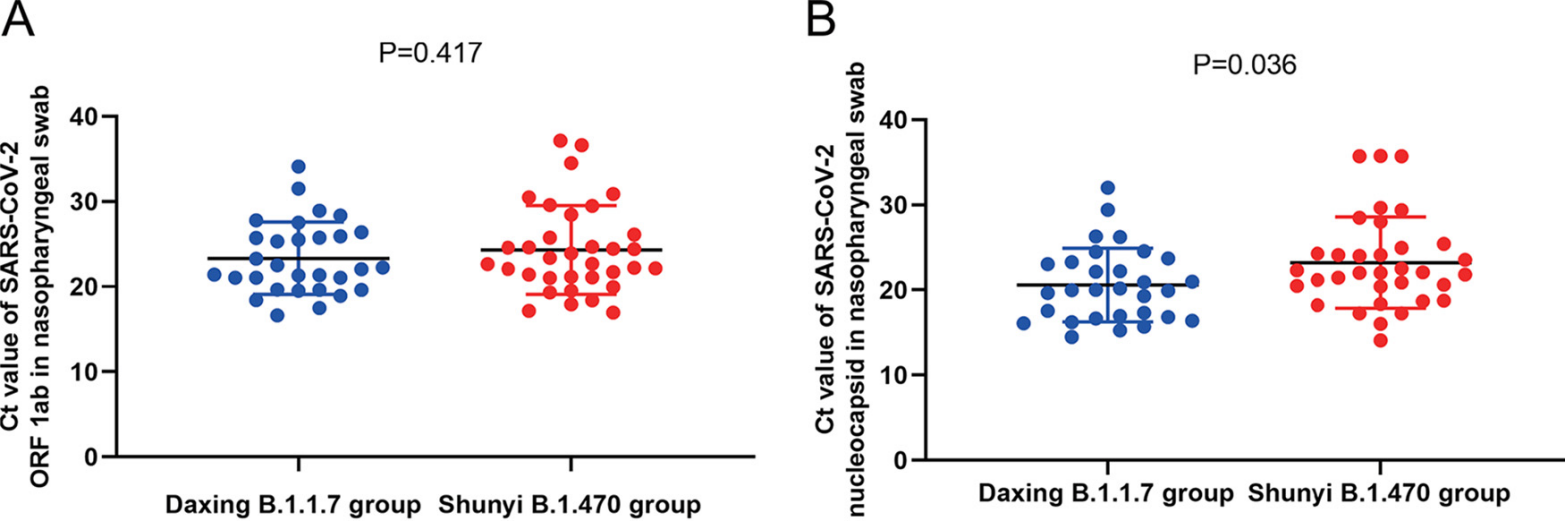
Scatterplot of the RT-qPCR *Ct* values of the Daxing B.1.1.7 group and the Shunyi B.1.470 group. (A) *C_t_* values of the OFR1ab gene target. (B) *C_t_* values of the N gene target. Samples from the Daxing B.1.1.7 group are shown as red dots, while those from the Shunyi B.1.470 group are shown as blue dots. Median *C_t_* is indicated by a black horizontal bar.

During the hospitalization of all the patients, several RT-qPCR tests using nasopharyngeal swabs were conducted regularly based on patients’ disease progression and clinical manifestations. The lowest threshold-crossing (*C_T_*) value of the ORF1ab and N genes of each patient was taken for comparison between the two groups (Table 4). The kits for RT-qPCR testing had the same lot numbers, and the instruments were the same. The median *C_T_* value of the ORF1ab gene target in the two groups was not significantly different, but that of the N gene target was significantly lower for the Daxing B.1.1.7 group than for the Shunyi B.1.470 group (*t* = 2.139, *P* = 0.036). The distribution of the samples in the two groups was compared within all ORF1ab and N gene *C_T_* values (Fig. 2). Figure 2B indicates that for the N gene, the median *C_T_* values of the Daxing B.1.1.7 group were higher. We also adjusted for the age factor and performed binary logistic regression analysis on the *C_T_* value (>18 or ≤18) (Table 5). Again, patients infected with the B.1.1.7 variant had a 4.484-fold higher risk of having N gene *C_T_* values of ≤18 in nasopharyngeal swab specimens than that of patients infected with nonvariants (OR = 4.484, *P* = 0.024) (Table 6).

**TABLE 4:** Risk of high CRP, high SAA, and GGO by variant (B.1.1.7 variant infection or non-B.1.1.7 variant infection)

Outcome	Variable	Regression coefficient	SE	Wald Chisq value	*P* value	Odds ratio	95% CI for OR
Level of CRP (<7 mg/liter or ≥7 mg/liter)	Age	0.00	0.01	0.03	0.854	1.00	0.98, 1.03
	Group	1.09	0.54	4.02	0.045	2.79	1.03, 0.86
Level of SAA (<10 mg/liter or ≥10 mg/liter)	Age	0.03	0.02	2.63	0.105	1.03	0.99, 1.06
	Group	1.62	0.63	6.54	0.011	5.03	1.45, 17.35
Had GGO or not	Age	0.01	0.02	0.69	0.408	1.01	0.98, 1.04
	Group	1.69	0.61	7.80	0.005	5.42	1.66, 17.73
Level of CK (<150 U/liter or ≥150 U/liter)	Age	0.00	0.02	0.05	0.821	1.00	0.97, 1.04
	Group	−1.52	0.73	4.34	0.034	0.22	0.05, 0.91
Level of DD (<0.5 mg/liter or ≥0.5 mg/liter)	Age	0.06	0.03	6.14	0.013	1.07	1.01, 1.12
	Group	0.70	0.82	0.73	0.392	2.01	0.41, 10.02
Level of CD4^+^ T (<706 cells/μl or ≥706 cells/μl)	Age	0.05	0.02	7.55	0.006	1.05	1.01, 1.08
	Group	1.20	0.55	4.77	0.029	3.31	1.13, 9.71

**TABLE 5:** Lowest RT-qPCR *C_t_* values in the two groups

Gene target	Lowest *C_T_* values for Daxing B.1.1.7 group (*n* = 33)	Lowest *C_T_* values for Shunyi B.1.470 group (*n* = 41)	*P* value
ORF1ab (mean ± SD)	23.33 ± 4.24	24.31 ± 5.21	0.417
N (mean ± SD)	20.58 ± 4.34	23.22 ± 5.37	0.036

**TABLE 6:** Risk of *C_t_* value of ≤18 or >18 of N gene target by variant (B.1.1.7 variant infection or non-B.1.1.7 variant infection)

Variable	Regression coefficient	SE	Wald Chisq value	*P* value	Odds ratio	95% CI for OR
Age	−0.01	0.02	0.42	0.518	0.99	0.96, 1.02
Group	1.50	0.67	5.08	0.024	4.48	1.22, 16.53

## DISCUSSION

Numerous studies have indicated that the SARS-CoV-2 B.1.1.7 variant has transmissibility higher than that of the nonvariant, but whether it has increased pathogenicity remains controversial. In this study, we analyzed the differences in clinical characteristics, laboratory tests, RT-qPCR results, and whole-genome features between 33 patients infected with the SARS-CoV-2 B.1.1.7 variant and 41 infected with the nonvariant. The results indicated that COVID-19 patients infected with the B.1.1.7 variant had more serious clinical features and possibly higher viral loads. This implies that the B.1.1.7 variant may have increased pathogenicity.

In the Daxing B.1.1.7 group, we observed patients older than those in the Shunyi B.1.470 group, probably due to the living characteristics of residents from the community, as many retired older people were more frequently active in the community.

B.1.1.7 variant infection could lead to a more serious inflammatory response, an acute response process, and more severe pneumonia, as indicated by the following results: in the B.1.1.7 variant cases, (i) there were more fevers over 38°C, (ii) CRP, SAA, CK, and DD were significantly higher, (iii) an abnormal proportion of CD4^+^ T was significantly more common, and (iv) ground-glass opacity in the lung was significantly more common.

Comparing the *C_T_* values of the RT-qPCR results between the two groups, samples from the Daxing B.1.1.7 group had lower *C_T_* values of the N gene, from which it could be speculated that B.1.1.7 variant samples may have a higher viral load. This result is in line with previous research showing that the B.1.1.7 variant is associated with significantly higher viral loads in samples tested by ThermoFisher TaqPath RT-qPCR (14). Even though the sample size in this study was relatively small and no significant difference was detected in the open reading frame (ORF) gene, the statistical results for the N gene could still indicate a higher infectivity of the B.1.1.7 variant.

SARS-CoV-2 B.1.1.7 variant infection may be the main risk factor for more serious clinical features after adjusting for age. Old age has been considered an important factor in SARS-CoV-2 infection and severe COVID-19 because elderly patients have weaker immune systems and are prone to multisystem organ dysfunction and even failure (15–18). Thus, we adjusted for the older age of the Daxing B.1.1.7 group and still found that cases infected with the B.1.1.7 variant presented more serious clinical features and higher infectivity.

The phylogenetic analysis showed that the strains from the two groups belonged to different lineages. The whole-genome analysis revealed that the case samples from group DX B.1.1.7 had 3 to 4 specific substitutions in addition to the 28 nucleotide substitutions corresponding to the B.1.1.7 reference sequence, which indicated that the strains might have been evolving for some time before being transmitted to China. Furthermore, only two amino acid mutations in the S protein were detected in the strains from the Shunyi outbreak, which suggests transmissibility lower than that of the B.1.1.7 strains.

Currently, there is no direct biological experimental evidence that can confirm the increased pathogenicity or virulence of the B.1.1.7 variant, and the clinical characteristics of B.1.1.7 variant infection have rarely been assessed. This prospective, comparative cohort study of COVID-19 cases from two COVID-19 outbreaks in Beijing implied that COVID-19 cases caused by the B.1.1.7 variant presented clinical and laboratory characteristics more serious than those of cases caused by the non-B.1.1.7 variant (lineage B.1.470).

This study has limitations. First, due to the very low morbidity of COVID-19 now in China, the sample size in the two groups of this study was relatively small. Second, we were not able to compare the clinical features between the cases infected with the B.1.1.7 variant and the cases infected with SARS-CoV-2 lineages other than B.1.470. Because during December 2020 to January 2021 only these two clustered outbreaks had taken place in Beijing, the data of these two groups were more comparable than other groups of data would be.

## MATERIALS AND METHODS

### Ethical approval.

The study was approved by the Institutional Review Board of Beijing Ditan Hospital, Capital Medical University in Beijing (approval number JDLY2020-020-01).

### Study design and recruitment of cases.

This study included a total of 74 confirmed COVID-19 cases from Beijing Ditan Hospital, which were divided into the Daxing B.1.1.7 group and the Shunyi B.1.470 group. The data of all the cases from the two groups were extracted from the cloud database. The confirmed cases referred to patients with clinical features of COVID-19 and a positive RT-qPCR test for SARS-CoV-2 RNA at least once in the respiratory specimens collected. The Daxing B.1.1.7 group contained a total of 33 cases, whereas the Shunyi B.1.470 group contained 41 cases. COVID-19 patients were diagnosed according to the 8th version of *Diagnosis and Treatment Protocol for Novel Coronavirus Pneumonia Patients*, and the clinical severity was categorized into 4 grades: mild (the clinical symptoms were mild, and there was no sign of pneumonia on imaging), moderate (fever and respiratory symptoms, imaging manifestations of pneumonia), severe (dyspnea with a respiratory rate of >30/min, hypoxemia with oxygen saturation of <93%, PaO2/FiO2 of <300 mm Hg, or the clinical symptoms worsened gradually, and the lung imaging showed that the lesions progressed more than 50% within 24 to 48 h), and critical (developed complications, including respiratory failure that needed mechanical ventilation, shock, or other organ failure that needed intensive care unit [ICU] monitoring treatment). Asymptomatic patients with no clinical symptoms but positive RT-qPCR results were also admitted to the hospital. Each patient was observed for at least 28 days. As of this study (June 18, 2021), all the patients had been discharged.

### Data collection.

The data of the 74 COVID-19 cases from the two groups were extracted from the cloud database. Medical record review was performed to collect patients’ underlying medical conditions and symptoms at the time of diagnosis, including data on the chronology of symptom onset, history of first presentation, disease progression, past medical history, physical findings, laboratory test results, imaging results, treatment, and hospital course. In addition, epidemiological data of the patients were collected. Individual data were compiled into two groups of patients, those with the Daxing B.1.1.7 group and those with the Shunyi B.1.470 group, for further analyses.

### Laboratory testing.

Oropharyngeal swabs, nasopharyngeal swabs, or sputum specimens obtained from patients during their hospital stays were collected for RT-qPCR testing. Viral RNA was extracted directly from 200-μl swab samples with a QIAamp viral RNA minikit (Qiagen, Germany). RT-qPCR was conducted using the novel SARS-CoV-2 nucleic acid test kit (BioGerm, Shanghai, China) with a fluorescence PCR detector following the manufacturers’ instructions. A TaqMan probe-based kit was designed to detect the ORF1ab and N genes of SARS-CoV-2 in 1 reaction. Corresponding serum samples were tested for anti-SARS-CoV-2 antibodies using a chemiluminescence immunoassay (CLIA; Bioscience, Chongqing, China).

### Whole-genome sequencing and analysis.

The selected swab samples of the patients were then sent to the Chinese CDC for further whole-genome sequencing. Libraries were prepared using a Nextera XT library prep kit (Illumina, San Diego, CA, USA), and the resulting DNA libraries were sequenced on either a MiSeq or an iSeq platform (Illumina) using a 300-cycle reagent kit. Mapped assemblies were generated using the SARS-CoV-2 genome (accession number NC_045512) as a reference. Variant calling, genome alignment, and sequence illustrations were generated with CLCBio software. Whole-genome sequence alignment was conducted using the Muscle tool in MEGA (version 7.0). A neighbor-joining phylogenetic tree was constructed using MEGA (version 7.0), and the Kimura 2-parameter model with 1,000 bootstrap replicates was used. Genomic lineage designation was performed using the PANGO lineage typing method (https://cov-lineages.org/).

### Statistical analysis.

The statistical analyses were performed using SPSS version 24.0 (SPSS IBM, Armonk, NY, USA). From each individual patient, we entered the demographic and clinical variables. Normal continuous variables are represented by mean and standard deviation. Student’s *t* test was used to find significant differences. Nonnormally distributed continuous variables are represented by median and quartile range, and the Mann-Whitney U test was used for comparison. Categorical variables are expressed as numbers and percentages, and the chi-squared test and Fisher’s exact test were used to compare them. Binary logistic regression analysis was performed to analyze the risk factors for the severity of COVID-19. All tests were two-tailed, and statistical significance was defined as a *P* value lower than 0.05.

### Data sharing.

The sequence data generated in this study are made available to researchers through the GISAID database (accession no. EPI_ISL_1121993 and EPI_ISL_1122015 to 1122017).
